# An Ionic Supported Liquid Membrane for the Recovery of Bisphenol A from Aqueous Solution

**DOI:** 10.3390/membranes12090869

**Published:** 2022-09-08

**Authors:** Manal Aldwaish, Noura Kouki, Azizah Algreiby, Haja Tar, Rafik Tayeb, Amor Hafiane

**Affiliations:** 1 Chemistry Department, College of Science, Qassim University, Buraydah 51452, Saudi Arabia; 2 Laboratory of Water, Membranes and Environment Biotechnology (EMBE), Technopole of Borj Cedria (CERTE), Hammam Lif 2050, Tunisia; 3 Chemistry Department, College of Science, Imam Abdulrahman Bin Faisal University, Dammam 34212, Saudi Arabia

**Keywords:** liquid membrane, bisphenol A, ionic liquid

## Abstract

In this work, a flat supported liquid membrane (FSLM) was applied for the extraction of bisphenol A (BPA) from aqueous solutions, using an ionic liquid as a carrier. The liquid membrane consists of tricaprylmethylammonium chloride (aliquat 336^®^) diluted in 2-octanol. Furthermore, to obtain the best transport efficiency, the impacts of various experimental parameters were investigated. These parameters included aliquat 336^®^ concentration, the concentration of BPA in the feed phase, the pH of the feed phase, the concentration of NaOH in the receiving phase, the polymeric support nature, the percentage of extractant in the organic phase, and the solvent nature. The optimum conditions of the experiment were 50% (*v*/*v*) aliquat 336^®^/2-octanol as the organic phase, a transport time of 8 h, and 1 × 10^−2^ mol L^−1^ NaOH as the receiving phase. The BPA was successfully recovered (the recovery percentage was about 89%). Supported liquid membrane-based aliquat 336^®^/2-octanol displayed an acceptable stability with re-impregnation after 5 days of operation.

## 1. Introduction

Bis(4-hydroxyphenyl)dimethyl methane, also known as bisphenol A (BPA), has been used in manufacturing as a significant intermediate in the production of a number of resins and polymers [1]. These products have been used in broad applications such as bottles, food packaging, toys, thermal paper, coatings, and medical supplies, either as a modified or polymerized chemical structure of BPA or via the use of BPA as an additive [2]. Bisphenol A is considered an endocrine-disrupting chemical (EDC); EDCs are defined as a structurally varied class of emerging pollutants that have been detected in aqueous environments, and can interfere with hormonal balance at low doses. This may affect the operation or function of the endocrine system in different ways, such as mimicking or blocking a natural hormone or causing the over- or underproduction of hormones [2,3]. The European Chemicals Agency classified BPA as a substance that may damage fertility, cause serious eye damage, cause an allergic skin reaction, and potentially cause respiratory irritation [4]. According to the European Food Safety Authority (EFSA), the temporary tolerable daily intake (t-TDI) for BPA is 4 μg kg^−1^ bw/day [5]. As a result of its harmful health effects and its carcinogenic nature, BPA should be removed from aqueous solutions.

Several analytical techniques have been used for the removal or degradation of BPA. I—Photocatalytic degradation [6,7,8] and biodegradation [9,10] are processes from which there is no mineralization, and biological processes result in a sludge discharge, causing an additional problem. II—Solvent extraction [11] is one of the most efficient separation methods, but this process is expensive and toxic due to the large quantities of organic solvents that are needed. III—The Fenton process, which produces OH· radicals, is one of the most important advanced oxidation processes used, due to its efficiency, cost-effectiveness, and environmental friendliness [12,13,14,15,16]. There are two drawbacks to the classic Fenton process: the need for very acidic pH values, and the production of secondary pollutants, such as iron sludge [17,18,19,20]. IV—Membrane technology is one of the methods applied for BPA extraction from aqueous media. Among the membrane-based separation processes, the use of liquid membranes has received growing attention. Panigrahi et al. used an SILM for the recovery of BPA from aqueous solution. For the SILM experiments, polyvinyldene fluoride (PVDF) membrane was used as a support medium, and ionic liquids based on different cations (e.g., phosphonium, imidazolium, ammonium, and pyridinium) were used as the membrane organic phase. Gupta et al. tested a hollow-fiber-supported liquid membrane (HFSLM) for the separation of bisphenol A from aqueous solution [21,22].

Supported liquid membrane (SLM) is a technology that merges the solvent extraction and re-extraction processes into one step. It is defined as an organic phase immobilized in the pores of an inert porous membrane (polymer or inorganic support material) by capillary forces placed among two aqueous solutions: the feed (source) and the strip (receiving) phases [23,24,25]. SLMs have several beneficial features, such as the requirement of minimal organic phase and extractant (carrier), the ability to achieve significant separation factors, and low operating cost. Problems with the stability and long-term performance of SLMs still limit their industrial application [26,27]. As conventional liquids evaporate, dissolve into a contacting phase, and displace from the porous structure, liquid membranes supported by conventional liquids eventually deteriorate [28].

Ionic liquids (ILs) involve organic and/or inorganic ions, and may contain more than one cation or anion [29]. Ionic liquids have been viewed as appropriate alternatives to conventional volatile organic solvents. Generally, ionic liquids possess attractive properties as solvents, such as chemical and thermal stability [30]. Moreover, the stabilization of SLMs could be enhanced by using ILs as a liquid phase [31,32]; this is attributed to their low vapor pressure, the possibility of reducing their solubility (by choosing the right cation and anion), and the greater capillary force due to their high viscosity [33,34]. The aim of this study was to remove BPA from an aqueous solution using a supported ionic liquid membrane (SILM). We investigated the parameters that may influence transport efficiency, such as the feed phase concentration, the pH of the feed phase, the receiving phase concentration, the percentage of extractant in the organic phase, solvent nature, and membrane stability.

## 2. Experimental

### 2.1. Materials

**Figure 1:**
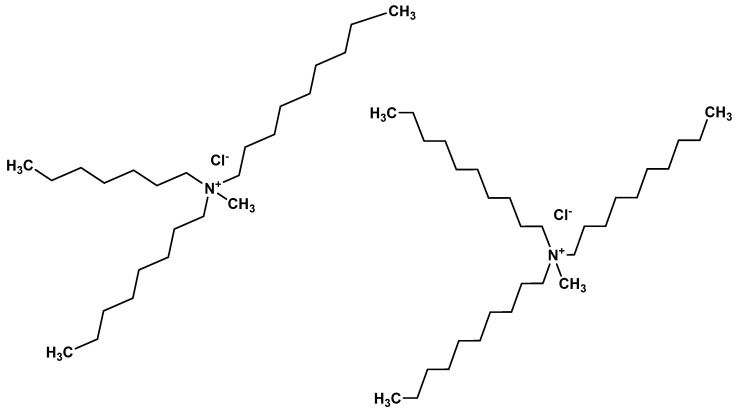
Aliquat^®^ 336 (tricaprylmethylammonium chloride).

**Figure 2:**
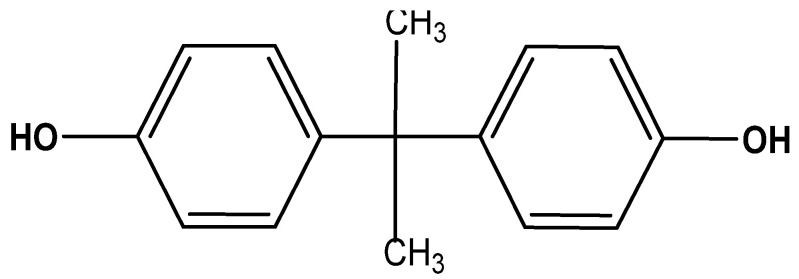
4,4′-(Propane-2,2-diyl) diphenol (bisphenol A).

Aliquat^®^ 336 (tricaprylmethylammonium chloride) is a mixture of C_8_ (octyl) and C_10_ (decyl) chains, with C_8_ predominating (Fluka, Wabash, IN, USA). The chemical structure of aliquat^®^ 336 is given in Figure 1. 2-Octanol (Riedel-de Haën, Wabash, IN, USA, 97%), nitrophenyl octyl ether (Fluka, Wabash, IN, USA, 99%), ethyl phenyl ketone (Alpha Chemika, Maharashtra, India. 98%), diphenyl ether (Riedel-de Haën, Wabash, IN, USA, 99%), dioxane (BDH, Dubai, United Arab Emirates, 99.7%), tetrahydrofuran (ACROS, Geel, Belgium 99%), benzyl methyl ketone (MERCK, Darmstadt, Germany, 98%), chloroform (LOBA CHEMIE, Mumbai, India, 99%), and cyclohexane (BDH, Dubai, United Arab Emirates, 99%) were used as organic solvents. Feed solutions were prepared by dissolving bisphenol A (molecular formula: C_15_H_16_O_2_; molecular mass: 228.261 g mol^−^^1^, ACROS, Geel, Belgium 97%) in ultrapure water (Milli Q Plus Colum, Millipore, Burlington, MA, USA). The chemical structure of BPA is given in Figure 2. The stripping agents were various salts: NaOH (CDH, New Delhi, India, 97%), NaCl (Pacegrove, Leicestershire, UK, 99%), sodium Acetate (LOBA CHEMIE, Mumbai, India, 99%), sodium iodate (Peking’s, Peking, China, 99.8%), and sodium benzoate (Oxford, Hartlepool, UK, 99%). Polypropylene (Accurel^®^ PP-2E Enka), polypropylene (Accurel^®^ PP 2E-HF; Membrana, Sunnyvale, CA, USA), polypropylene (Celgard 2500 Celgard Inc., Charlotte, NC, USA), and polyvinylidene difluoride (Durapore^®^ GVSP; Millipore, Burlington, MA, USA) were used as polymeric support for the organic solution.

### 2.2. Membrane Preparation

**Figure 3:**
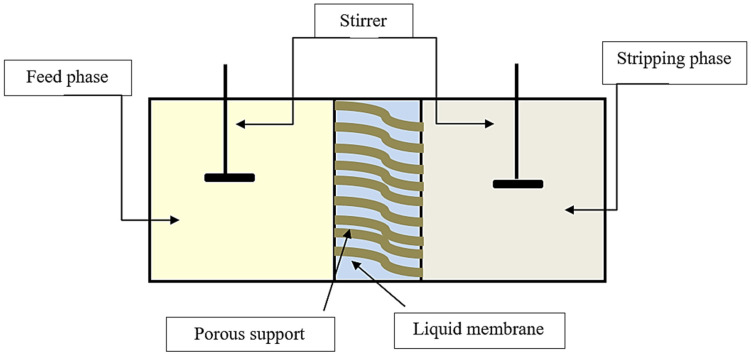
Schematic diagram of supported liquid membrane (SLM) process.

The membrane was saturated with ionic liquid for 24 h to prepare a flat-sheet supported liquid membrane (SLM). BPA extraction experiments were carried out at approximately 25 °C in a permeation cell that was in contact with an SLM membrane fixed between the two half-cells of the device, with an exposed membrane area of 3.14 cm^2^. The feed and stripping solutions (50 mL each) were separately delivered into two compartments located on the device and stirred using two magnetic stirrers. Figure 3 is a schematic illustration of the SLM process used during each experiment. In the transport operation, 0.5 mL of the feed and stripping solutions were extracted every hour using a pipette. These solution samples were then analyzed using a (Shimadzu UV-vis spectrophotometer 1650, Kyoto, Japan) at a wavelength of 276 nm.

The extraction rate E% and recovery rate R% were calculated as the following formula:

(1) $$E\%=\frac{{\left[BPA\right]}_{donor,0}-{\left[BPA\right]}_{donor,t}}{{\left[BPA\right]}_{donor,0}}\times 100$$

(2) $$R\%=\frac{{\left[BPA\right]}_{stripping,t}}{{\left[BPB\right]}_{donor,t}}\times 100$$

where ${\left[BPA\right]}_{donor,0}$ is the concentration of BPA in the initial feed solution, ${\left[BPA\right]}_{donor,t}$ is the concentration of BPA in the feed solution after transport, and ${\left[BPA\right]}_{stripping,t}$ is the concentration of BPA in the stripping phase after transport.

### 2.3. Buffer Solutions Preparation

A set of solutions of potassium chloride, hydrochloric acid, succinic acid, and glycine were prepared to adjust the pHs to 12, 10.6, 8.6, 6, 4, and 2.

The pHs of these solutions were maintained at different values ranging from 2 to 12 (Table 1). Mixtures of solutions of: potassium chloride (MERCK, Darmstadt, Germany, 99%) and hydrochloric acid; succinic acid (S D Fine-Chem Limited, Mumbai, India, 99%) and sodium hydroxide (CDH, New Delhi, India, 97%); and glycine (BDH, Dubai, United Arab Emirates, 99%) and sodium hydroxide (CDH, New Delhi, India, 97%) were prepared to obtain the pHs of 2, 4, 6, 8.6, 10.6, and 12.

## 3. Results and Discussion

### 3.1. Effect of Aliquat 336^®^ Percentage 

**Figure 4:**
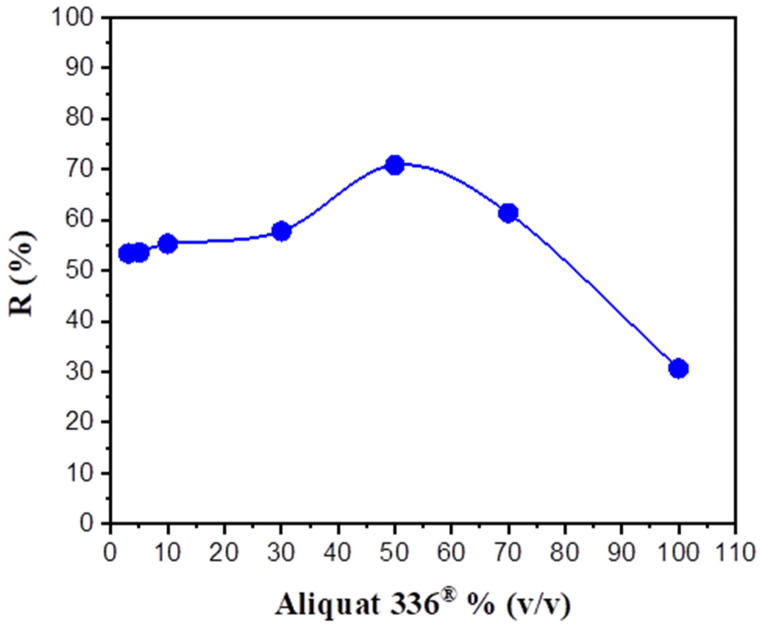
Effect of carrier concentration on the BPA recovery percentage. Feed solution: 100 mg L^−1^ BPA; polymeric support: Accurel^®^ 2E-PP; organic phase: various aliquat 336^®^ percentages in 2-octanol; receiving solution: 1 × 10^−2^ mol L^−1^ NaOH.

Transport experiments were conducted using membranes containing aliquat 336^®^/2-octanol to set the ideal combination for the extraction of BPA. Membranes with different amounts of aliquat 336^®^/2-octanol (3, 5, 10, 30, 50, 70, and 100% (*v*/*v*)) were prepared using Accurel^®^2E-PP. Figure 4 shows the change in the recovery rate in the receiving solution (R%) as a function of the aliquat 336^®^/2-octanol concentration after 8 h of the transport process. The transport of BPA increased with an increase in the aliquat 336^®^/2-octanol concentration from 3% to 50%, and then decreased. At an aliquat 336^®^/2-octanol concentration of 3%, the R% was 53.40%. With an increase in concentration, the peak value was acquired at an aliquat 336^®^/2-octanol concentration of 50% at R% (70.8%). With an increase in the carrier concentration up to 100%, R% decreased to 30.7%. 

Initially, the recovery percentage increased with carrier concentration. This increase could be related to the increased rate of complex formation at the interface on the feed side of the SLM, which may have resulted in an improvement of diffusion efficiency. However, the kinetic data began to decline when a certain limit value was exceeded because the BPA carrier complex formed at the donor–membrane interface was constrained in its diffusion due to the high viscosity of the membrane. This means that a higher concentration of aliquat 336^®^ would result in a lower transport rate for the solute [35,36,37].

### 3.2. Effect of BPA Concentration

**Figure 5:**
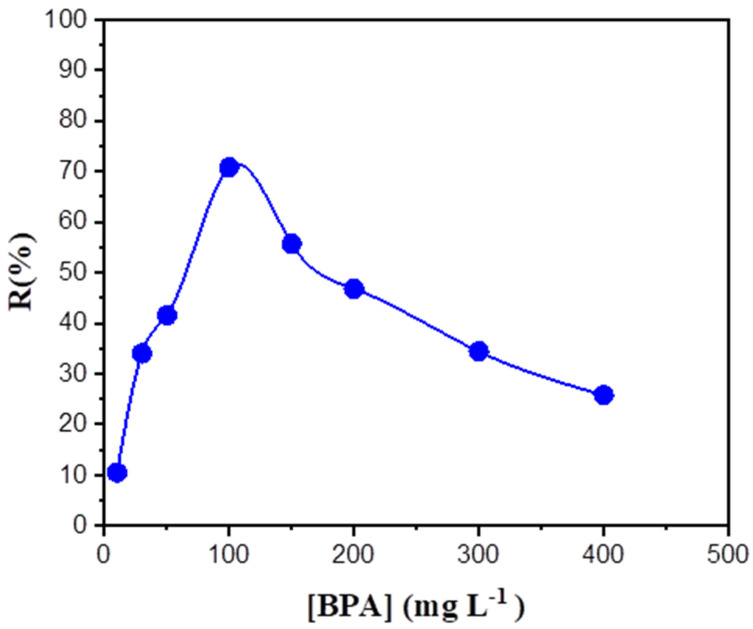
Effect of BPA concentration on the BPA recovery percentage. Feed solution: various concentrations of BPA; polymeric support: Accurel^®^ 2E-PP; organic phase: aliquat 336^®^ 50% (*v*/*v*) in 2-octanol; receiving solution: 1 × 10^−2^ mol L^−1^ NaOH.

This study was carried out using source solutions containing various BPA concentrations extending from 10 mg L^−1^ to 400 mg L^−1^. The results of the changes in recovery rate (%) are shown in Figure 5. With an increase in the BPA concentration from 10 mg L^−1^ to 100 mg L^−1^, the values of R% increased. For R%, this increased from 26.3% to 34%, 42.9%, and 70.8%, respectively. Thereafter, for the concentrations of BPA from 150 mg L^−1^ to 400 mg L^−1^, the values of R% decreased from 55.7% to 46.8%, 34.4%, and 25.8%, respectively. A low or constant concentration of BPA could be due to the saturation of membrane pores with BPA molecules and their accumulation on the membrane interface. In addition, it may be due to a lower effective area for transport. Therefore, increasing the contact area between the aqueous phase and the membrane phase can improve the separation efficiency [38,39].

### 3.3. Effect of NaOH Concentration 

**Figure 6:**
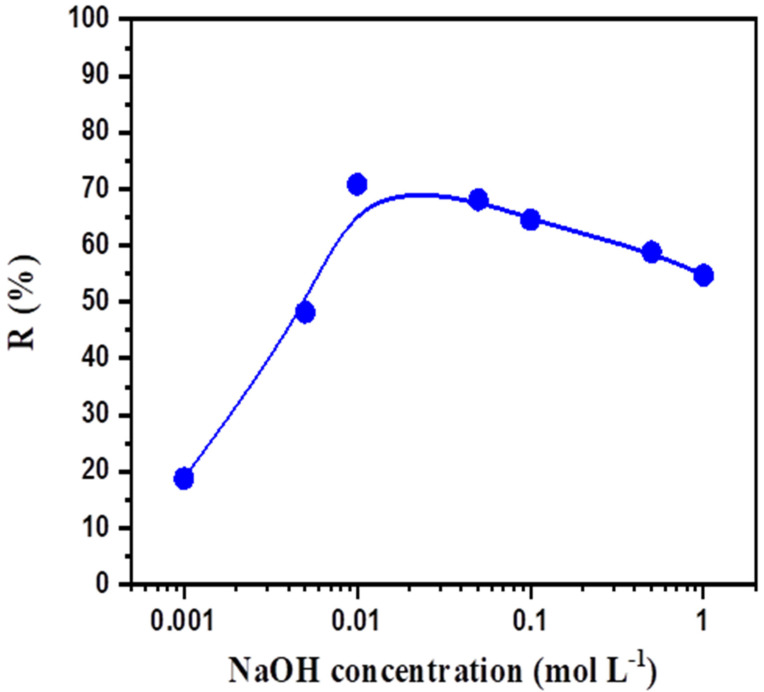
Effect of NaOH concentration on the BPA recovery percentage. Feed solution: 100 mg L^−1^ BPA; polymeric support: Accurel^®^ 2E-PP; organic phase: aliquat 336^®^ 50% (*v*/*v*) in 2-octanol; receiving solution: various concentrations of NaOH.

To assess the effect of NaOH concentration in the stripping solution on the transport of BPA, experiments were carried out by adjusting the NaOH concentration in the range from 1 × 10^−3^ mol L^−1^ to 1.0 mol L^−1^. As shown in Figure 6, with an increase in the NaOH concentration from 1 × 10^−3^ to 1 × 10^−2^ mol L^−1^, the values of R% increased from 18.8% to 70.8%, respectively. Thereafter, a variation in the concentration of NaOH from 5 × 10^−2^ mol L^−1^ to 1.0 mol L^−1^ decreased the values of R% from 68.1% to 54.7%, respectively. 

In the experiments that used 1 × 10^−3^ and 5 × 10^−3^ mol L^−1^, a lower final stripping efficiency was noticed. This could be due to the insufficiency of NaOH in the stripping phase. Theoretically, bisphenol reacts with NaOH in the stripping phase (after the diffusion process) to form sodium bisphenolate, while the activity of molecular bisphenol in the stripping phase is suppressed [40]. Thus, under a low concentration of NaOH, the activity of unreacted molecular phenol slowed the stripping process by reducing the concentration gradient between the membrane and stripping phases; hence, the stripping rate and efficiency were decreased. A very high concentration of NaOH yielded extreme alkalinity and increased the surface tension between the organic liquid and membrane filaments [41]. Nosrati et al. reported that a low concentration of NaOH is sufficient for the removal of BPA [42].

The stripping agent used to extract the BPA in this experiment was a 1 × 10^−2^ mol L^−1^ NaOH solution. In contrast, an extremely high NaOH concentration induced excessive alkalinity and increased the surface tension between the extractant and the membrane. This led to emulsification of the extractant, liquid membrane phase losses, and separation rate reduction. Similar results were observed by Meng et al. [43] for phenol transport through a polymer-inclusion membrane with N,N-di(1-methylheptyl) acetamide as the carrier from an aqueous solution, with a 1 × 10^−2^ mol L^−1^ NaOH solution used as the stripping phase to separate the phenolic compound. Thus, it is recommended that the concentration of NaOH not be very high or very low.

### 3.4. Effect of the Feed Phase pH

**Figure 7:**
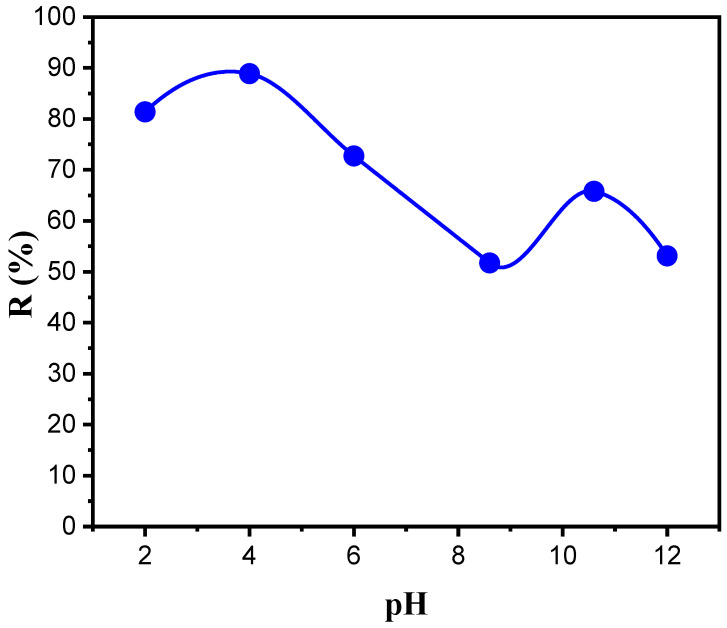
Effect of feed solution pH on the BPA recovery percentage. Feed solution: 100 mg L^−1^ BPA at different pHs; polymeric support: Accurel^®^ 2E-PP; organic phase: 50% (*v*/*v*) aliquat 336^®^ in 2-octanol; receiving solution: 1 × 10^−2^ mol L^−1^ NaOH.

The pH value of the sample significantly affects the BPA extraction procedure. The pH determines the degree of ionization and speciation of the analytes, which causes a different distribution of coefficients [44]. Figure 7 illustrates the influence of the changes in pH (from 2 to 12) of the feed solution on the transport of BPA. Obviously, an increase in the pH from 2 to 4 was useful for the transport of BPA across the membrane, leading to the highest values of R% (81.4% at pH 2 and 89% at pH 4). However, with a further increase in the pH, the values of R% decreased for pH values of 6, 8.6, 10.6, and 12 to 72.7%, 51.7%, 65.8%, and 53.1%, respectively. Therefore, the results suggest that the structure of the BPA molecule due to the acid–base equilibrium has a significant effect on transport.

**Figure 8:**
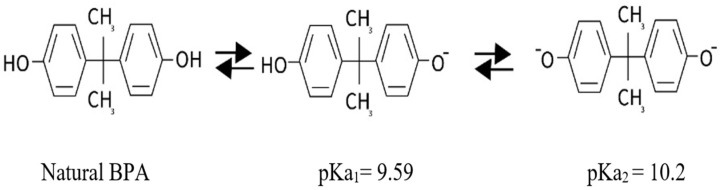
Different BPA forms in aqueous solution.

According to Staples et al. [45], there are two forms of BPA in solution: anion and dianion (Figure 8). Typically, neutral and ionic species exist in equal concentrations when pH = pKa. Moreover, neutral species dominate or ionic species dominate when pH < pKa or pH > pKa, respectively [46].

Therefore, it is recommended that the feed solution be adjusted to be acidic to ensure the existence of BPA in molecular form. Indeed, when the pH became slightly acidic or basic, the extraction distribution ratio decreased greatly due to BPA dissociation (pKa_1_ = 9.59 and pKa_2_ = 10.2) [47]. A pH of 4 was chosen as the best pH for further experiments [48].

**Figure 9:**
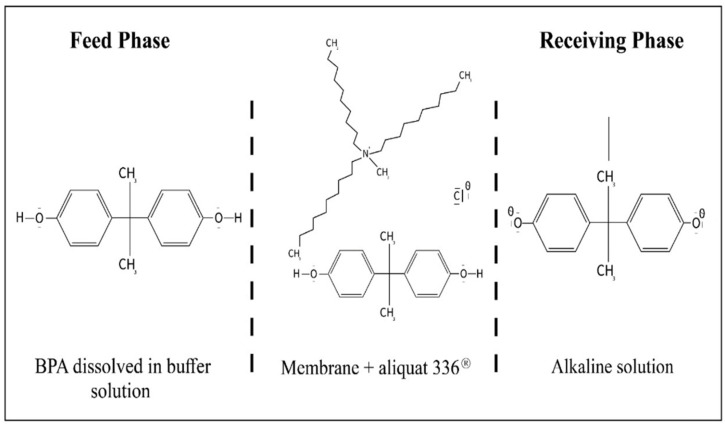
Transport mechanism of BPA through the SILM.

The transport of BPA through the SLM (Figure 9) can be described in three steps:

Firstly, at the interface (i.e., source phase–membrane) there is a solvation of BPA with R_4_N^+^, followed by ion pair formation (BPA). (R_4_N)+ Cl^−^. Secondly, the ion pair diffuses through the membrane and reaches the membrane-stripping phase interface. Finally, BPA is stripped on contact with NaOH.

BPA + (R_4_NCl)org → (BPA). (R_4_N) + Cl^−^

(BPA). (R_4_N)^+^ Cl^−^ + (Na^+^, OH^−^) → (R_4_NCl)org + BPA^−^Na^+^ + H_2_O

### 3.5. Effect of the Diluent

**Figure 10:**
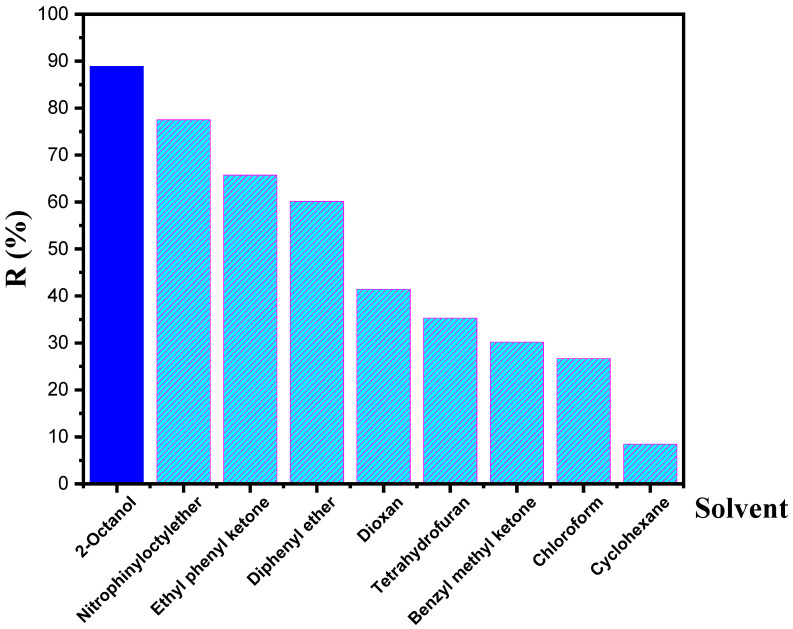
Effect of the diluent nature on the BPA recovery percentage. Feed solution: 100 mg L^−1^ BPA at pH 4; polymeric support: Accurel^®^ 2E-PP; organic phase: 50% (*v*/*v*) aliquat 336^®^ in various diluents; receiving solution: 1 × 10^−2^ mol L^−1^ NaOH.

Ion transport is affected by the physical and chemical properties of the diluents. Moreover, the significant factor for selecting diluents is membrane stability, fast transport, and the polarity of diluents [49]. Table 2 illustrates several properties of diluents used. The impacts of different solvents—2-octanol, nitrophenyl octyl ether, ethyl phenyl ketone, diphenyl ether, dioxane, tetrahydrofuran, benzyl methyl ketone, chloroform, and cyclohexane—were examined to identify their recovery efficiencies. Figure 10 shows that 2-octanol presented a higher recovery efficiency, which is attributed to the high polarity of 2-octanol. The maximum recovery yield was observed as follows: 89% for 2-octanol, 77.5% for nitrophenyl octyl ether, 65.7% for ethyl phenyl ketone, 60.1% for diphenyl ether, 41.4% for dioxane, 35.2% for tetrahydrofuran, 30.1% for benzyl methyl ketone, 26.7% for chloroform, and 8.4% for cyclohexane.

### 3.6. Nature of the Stripping Agent

There have been several reports about the influence of the stripping agent on the performance of the SILM process, where the receiving phase and its concentration have an important role in determining the final recovery of the target solute when using the SILM technique [50].

**Figure 11:**
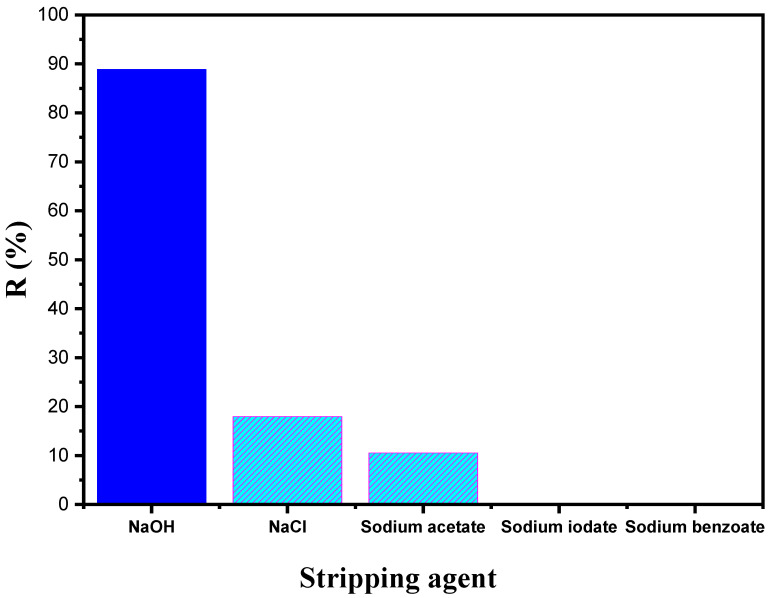
Effect of the stripping agent nature on the BPA recovery percentage. Feed solution: 100 mg L^−1^ BPA at pH 4; polymeric support: Accurel^®^ 2E-PP; organic phase: 50% (*v*/*v*) aliquat 336^®^ in 2-octanol; receiving solution: 1 × 10^−2^ mol L^−1^ of various stripping agents.

For transport studies in SLMs, the selection of an adequate stripping agent and its optimal concentration play a part in efficient function. For this reason, different species of stripping agents were investigated as an aqueous stripping phase. Figure 11 shows that the recovery percentage was 89% for sodium hydroxide, 18% for sodium chloride, and 10.5% for sodium acetate. However, with sodium iodate and sodium benzoate, there is no recovery value. NaOH was found to be best among the stripping agents. Rosly et al. reported the ability of a strong base at a lower concentration to strip phenol molecules from the organic phase through interaction with it [51].

The variation in pH occurring between the feed and receiving phases could be one of the reasons for transport of BPA through SILM. Compared to salts such as NaCl (pH = 7), CH_3_COONa (pH = 5), NaIO_3_ (pH = 11), and C_6_H_5_COONa (pH = 8.1), NaOH (pH: 14) is a strong base with a high dissociation constant. Thus, it results in the effective complexation of BPA with NaOH [52].

### 3.7. Effect of the Polymeric Support Type

Under identical experimental conditions, four polymeric supports were examined: polypropylene (Accurel^®^ PP-2E Enka), polypropylene (Accurel^®^ PP 2E-HF; Membrana, Sunnyvale, CA, USA), polypropylene (Celgard 2500 Celgard Inc., Charlotte, NC, USA), and polyvinylidene difluoride (Durapore^®^ GVSP; Millipore, Burlington, MA, USA). A solution of 1 × 10^−2^ mol L^−1^ NaOH was used as the stripping phase, a solution of 100 ppm BPA was dissolved in ultra-pure water as the feed phase, and 50% (*v*/*v*) aliquat 336^®^/2-octanol was used as the liquid membrane.

The experimental (J_exp_) and normalized (J_N_) BPA fluxes offered in Table 3 that were obtained with Durapore (in relation to the thickness $d_{0,A\;}$, porosity $\epsilon_{A}$, and tortuosity $\tau_{A}$ of the Accurel^®^ PP support) are described using the following Equation (3) [24,25]:

(3) $$J_{N}\;=J_{\exp}\;\frac{d_{0}\tau }{\epsilon }\;\frac{\epsilon_{A}}{d_{0,A\;}\tau_{A}}$$

where $d_{0}$, τ, and ϵ represent the membrane thickness, tortuosity, and porosity, respectively. The normalized flux values of BPA were compared to those obtained for salicylic acid [53] and acetaminophen [54] using polypropylene (Accurel^®^ PP 2E-HF) and polyvinylidene difluoride as the polymeric support.

**Figure 12:**
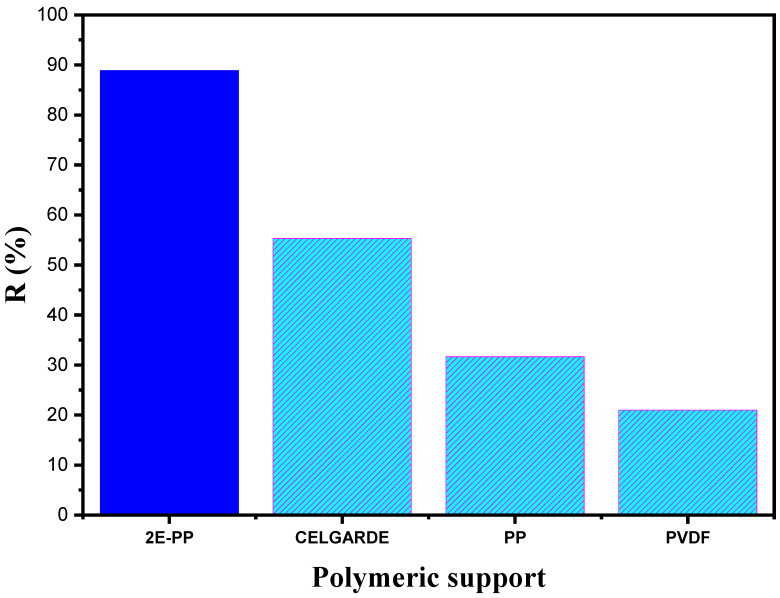
Effect of the membrane types on the BPA recovery percentage. Feed solution: 100 mg L^−1^ BPA at pH 4; polymeric support: set of membranes; organic phase: 50% (*v*/*v*) aliquat 336^®^ in 2-octanol; receiving solution: 1 × 10^−2^ mol L^−1^ NaOH.

The results illustrated in Figure 12 proved that the polymeric support 2E-PP displayed the best performance for the transport of BPA, with an acceptable R% value of 89%. Additionally, the polymeric support PP and PVDF exhibited lower values of BPA that were recovered in the stripping phase after 8 h of transport. The R% values were 31.66% for PP and 21% for PVDF. Using a Celgard membrane, the recovery efficiency of BPA was higher than those of the PP and PVDF membranes, and was equal to 55.31%.

This result does not match the physical parameters (thickness, porosity, tortuosity) of each support, since Celgard 2500 would normally give the highest flux due to its higher $\epsilon /d_{0}\tau$ ratio. This disagreement indicates that both the physical parameters and the chemical composition affect the transport efficiency [55,56,57].

(4) $$J_{\exp}=(\frac{V}{A})\;\left(\frac{dC}{dt}\right)$$

where V, A, C, and t represent the volume of the receiving phase (l), the active area of the membrane (m^2^), the concentration of BPA (mol L^−1^), and the transport time (s), respectively. $\frac{dC}{dt}$ is the slope calculated from the linear variation of the BPA concentration in the receiving phase versus time.

### 3.8. Membrane Stability

**Figure 13:**
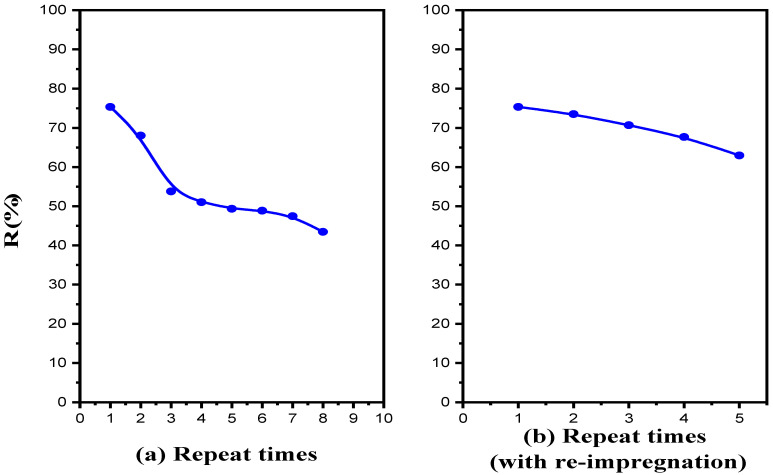
(**a**). Effect of the membrane stability on the BPA recovery percentage. Feed solution: 100 mg L^−1^ BPA at pH 4; polymeric support: Accurel^®^ 2E-PP; organic phase: 50% (*v*/*v*) aliquat 336^®^ in 2-octanol; receiving solution: 1 × 10^−2^ mol L^−1^ NaOH. (**b**) Effect of re-impregnation of the membrane used on the BPA recovery percentage. Feed solution: 100 mg L^−1^ BPA at pH 4; polymeric support: Accurel^®^ 2E-PP; organic phase: 50% (*v*/*v*) aliquat 336^®^ in 2-octanol; receiving solution: 1 × 10^−2^ mol L^−1^ NaOH.

To assess membrane stability, transport experiments were carried out in a continuous manner under the optimum conditions by reusing the same membrane for eight cycles. The recovery percentage of BPA was determined every 8 h. After each cycle, the feed and stripping solutions were replenished with new solutions. Figure 13a shows that the R% of BPA gradually decreased from 89% to 43.5% over eight continuous cycles (3 days of operation). This decline could be related to the loss of the liquid membrane. After the eighth cycle, the same membrane was used with re-impregnation to each cycle under the same conditions for five cycles (5 days). The recovery percentage of BPA was determined every 8 h. The results in Figure 13b reveal that the transport efficiency was slightly down, from 75.3% to 63%, over five cycles. The improvement in transport efficiency confirms the loss of the liquid membrane. Consequently, the results of re-impregnation show the possibility of reuse for the same polymeric support. 

## 4. Conclusions

The main objective of the present work was to recover BPA from an aqueous solution using SILM. Our investigation of the transport of bisphenol A from the feed solution to the stripping solution through the developed system showed satisfactory transport efficiency after the addition of 2-octanol as a diluent to aliquat 336^®^ in the membrane organic phase. The nature and the concentration of the stripping agent in the receiving phase, the diluent nature, and the carrier concentration in the membrane phase were optimized. Moreover, in the current study, under optimal experimental conditions (50% (*v*/*v*) aliquat 336^®^/2-octanol, a transport time of 8 h, 100 mg L^−1^ BPA at pH 4, and 1 × 10^−2^ mol L^−1^ NaOH as the receiving phase), the membrane stability test revealed a remarkable decrease in R% due to the loss of the liquid membrane. This decrease was improved by the re-impregnation of the used polymeric support. The developed system can be viewed as a good method for the recuperation of BPA from aqueous solutions. It is easy to implant and displays a very high performance (a recovery efficiency of approximately 89% was obtained). The obtained results will be very useful for a future study dealing with a mathematical model and characterization of the elaborated system.

## Figures and Tables

**Table 1:** Preparation of buffers.

The pH Buffer	Method of Preparation
pH 12	Glycine–NaOH buffer Stock solution: A: 0.2 M solution of glycine (1.5 g in 100 mL) B: 0.2 M NaOH 50 mL of A + 46.5 mL of B, diluted to a total of 200 mL
pH 10.6	Glycine–NaOH buffer Stock solution: A: 0.2 M solution of glycine (1.5 g in 100 mL) B: 0.2 M NaOH 50 mL of A + 45.5 mL of B, diluted to a total of 200 mL
pH 8.6	Glycine–NaOH buffer Stock solution: A: 0.2 M solution of glycine (1.5 g in 100 mL) B: 0.2 M NaOH 50 mL of A + 4 mL of B, diluted to a total of 200 mL
pH 6	Succinic buffer Stock solution: A: 0.2 M solution of succinic acid (5.9 g in 250 mL) B: 0.2 M NaOH 25 mL of A + 43.5 mL of B, diluted to a total of 100 mL
pH 4	Succinic buffer Stock solution: A: 0.2 M solution of succinic acid (5.9 g in 250 mL) B: 0.2 M NaOH. 25 mL of A + 10 mL of B, diluted to a total of 100 mL
pH 2	Hydrochloric acid–Potassium chloride buffer Stock solution: A: 0.2 M solution of KCl (7.46 g in 50 mL) B: 0.2 M HCl. 50 mL of A + 10.6 mL of B, diluted to a total of 200 mL

**Table 2:** Formula, viscosity, and dielectric constant of organic solvents.

Compound	Formula	Viscosity η (cP)	Dielectric Constant ɛ_r_
Tetrahydrofuran	C_4_H_8_O	0.55	7.58
Chloroform	CHCl_3_	0.57	4.81
Cyclohexane	C_6_H_12_	1	18.5
Dioxane	C_4_H_8_O	1.54	2.21
Diphenylether	C_12_H_10_O	3.9	-
2-Octanol	C_8_H_18_O	10.6	3.4
Nitrophenyloctylether	C_14_H_21_NO_3_	12.8	23.1
Benzylmethylketone	C_9_H_10_O	-	-
Ethylphenylketone	C_9_H_10_O	-	-

**Table 3:** Physical characteristics of polymeric supports, as well as experimental (J_exp_) and normalized fluxes (J_N_) of BPA.

Polymeric Support	Durapore® GVSP Millipore, USA	Celgard 2500 Celgard Inc., USA	Accurel^®^ PP-2E Enka	Accurel® PP 2E-HF Membrana, USA
Material	Polyvinylidene Difluoride	Polypropylene	Polypropylene	Polypropylene
**Thickness d_0_ (** **μm)**	120	25	130–180	160
**Pore diameter d (μm)**	0.2	0.064	0.2	0.2
**Porosity ɛ (%)**	65	55	70	75
**Tortuosity (τ = 1 − lnε)**	1.43	1.598	1.357	1.29
**ε/d_0_τ (10^−3^ µm^−1^)**	3.788	13.76	3.328	3.633
**J_exp_ (10^−6^ mol m^−2^ s^−1^)**	4.8	0.32	0.48	0.16
**J_N_ (10^−6^ mol m^−2^ s^−1^)**	4.6	0.084	0.524	0.16
**J_N_ (10^−6^ mol m^−2^ s^−1^)**	8.6 [53]			6.9 [53]
**J_N_ (10^−6^ mol m^−2^ s^−1^)**	0.43 [54]			4 [54]

## Data Availability

Not applicable.
